# Advice for smokers in smoking cessation clinic: a review

**DOI:** 10.4314/ahs.v23i2.42

**Published:** 2023-06

**Authors:** Xuechan Yu, Meihua Wang, Jie cen, Mianzhi Ye, Sha Li, Younuo Wang, Qingwen Su, Hui Chen, Ruyi Xu, Shuya Zhang, Shanshan Wang, Yiming Yu, Zaichun Deng, Zhongbo Chen

**Affiliations:** 1 Department of Pulmonary and Critical Care Medicine, The First Affiliated Hospital of Ningbo University No.247, Renmin Road, Jiangbei District Ningbo, Zhejiang Province, China, 315010; 2 Department of Neurology, Ningbo ninth Hospital No.68, Xiangbei Road, Jiangbei District Ningbo, Zhejiang Province, China, 315010; 3 Department of Pulmonary and Critical Care Medicine, Ningbo ninth Hospital, No.68, Xiangbei Road, Jiangbei District Ningbo, Zhejiang Province, China, 315010; 4 Department of Prevention and Health Care, The First Affiliated Hospital of Ningbo University No.247, Renmin Road, Jiangbei District Ningbo, Zhejiang Province, China, 315020

**Keywords:** Tobacco dependence, smoking cessation, risk factors, advice, addiction

## Abstract

**Background:**

Tobacco dependence has become a global public health concern. We chose to investigate the modifiable factors and motivations during the period of smoking cessation based on the mechanism of nicotine addiction.

**Methods:**

We selected emotion, sleep, alcohol, caffeine beverages, mental activities after dinner, exercise and CYP2A6 genotype as influencing factors, and provided corresponding recommendations for smokers based on these factors. Based on these characteristics, we reviewed literature and summarized the relationship between these factors and nicotine dependence or smoking.

**Results:**

Different emotion, sleep deficiency, caffeine intake, alcohol consumption, mental activities after dinner, physical exercises and CYP2A6 genotype have an effect on daily smoking and nicotine dependence.

**Conclusion:**

These suggestions related literature-derived factors may increase the success rate of smoking cessation.

## Introduction

Tobacco dependence has become a global public health concern^1^. As a result of the efforts of doctors around the world and the development of tobacco control policies^2^-^4^, a growing number of smokers are becoming more aware of the benefits of quitting. However, nicotine withdrawal symptoms made it difficult for these smokers to quit^5^,^6^, so they began to visit the hospital's smoking cessation clinic for assistance. A After nicotine binds to nicotinic acetylcholine receptors (nAChRs) α4β2 receptors, dopamine is produced, which alters the firing of dopamine neurons in response to reinforcement^7^. As large quantities of dopamine continue to be released, the firing mode of dopamine neurons changes from reinforcement to hijack, resulting in addiction^7^. Numerous studies have demonstrated the impact of life behaviours or motivations on cigarette consumption and nicotine dependence 8, 9, such as working time^10^, socioeconomic pressure^11^, caffein exposure^12^, alcohol^7^, sleeping time^13^, ^14^, *CYP2A6* gene type^15^, etc. Nowadays, there are many treatment options and methods for nicotine addiction^16^. Changes in life behaviour or smoking motivations played a crucial role in smoking cessation, regardless of the type of treatment^9^,^16^. However, not all factors and behaviours can be altered during smoking cessation therapy. In order to increase the success rate, cessation clinics should inquire about each smoker's daily habits and smoking motivations in order to formulate counselling suggestions for smokers during their quitting period. Here are some modifiable factors and advice that help smokers without mental disorders successfully quit.

## Methods

Smokers who visited our clinic from December 2008 to December 2021 at the Affiliated Hospital of Medical School of Ningbo University exhibited the following common smoking characteristics. (1) Negative and positive emotions; (2) Sleep deficiency; (3) Alcohol intake; (4) Caffeine drink intake; (5) Mental activities after dinner prior to sleep; (6) Physical exercise; (7) CYP2A6 genotypes. We reviewed the literature according to these characteristics and summarized the relationship between these characteristics and nicotine addiction or smoking. On the basis of these mechanisms and the unique circumstances of each smoker, recommendations are made to optimize individualized treatment plans, thereby reducing the difficulty of quitting and increasing the success rate.

### Negative and positive emotions

Different smokers may choose to light a cigarette based on their mood at the time. Russell's questionnaire^17^ divided questions about emotional motives into two categories: enjoyment and calm. During the smoking cessation period, these questions are used to formulate advice for smokers who are attempting to quit. Dopamine actually processes both rewarding and aversive stimuli 18. he ventral tegmental area (VTA) encodes the event with dopamine concentration^19^, ^20^. When a smoker experiences positive or negative emotions after being exposed to external stimuli. When positive emotions appear, the brain responds by producing large quantities of dopamine in the striatal regions. In contrast, when negative emotions appear, two distinct populations of dopamine neurons in mesencephalic dopamine cells continue to produce dopamine to deal with them^21^. Due to differences in neuronal count, the amount of dopamine produced during different emotions varies 7. Clearly, a greater concentration of dopamine is secreted during positive emotions. based on the discharge characteristics of dopamine neurons, smoking during positive emotions is more likely to result in dependence than smoking during negative emotions (Picture 1). We can speculate that smokers did not choose smoking as a means of coping with their emotions.

**Figure:**
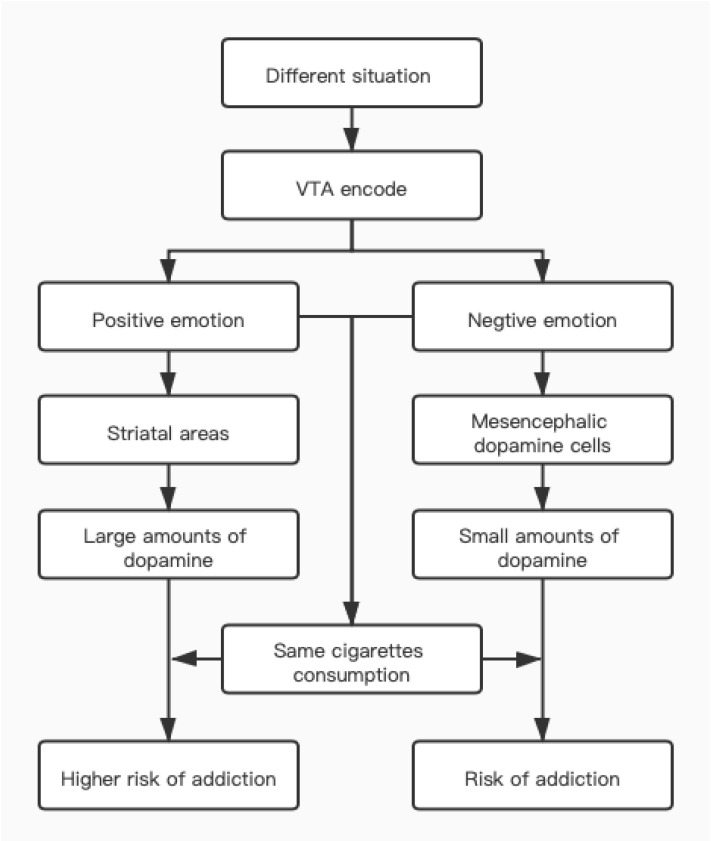
Picture 1

### Sleep deficiency

Some smokers have the bad habit of staying up at night for a long time and they often smoke at night before sleep. Smoking disrupts sleep latency, sleep duration and sleep quality^14^. This is why sleep deficiency is more prevalent among smokers than among non-smokers. Attention, decision-making, and executive functioning degrade significantly after long periods of wakefulness,^22^-^24^ so dopamine from tobacco can improve these abilities. Smokers with inadequate sleep duration or quality are associated with higher levels of withdrawal, craving, and smoking urges 13, 25, which makes quitting smoking difficult. Therefore, quitting smoking at night improves sleep duration and quality, reducing cigarette consumption and nicotine dependence.

### Alcohol intake

Due to the synergistic effects of nicotine and ethanol on VTA DA function in reinforcement^7^, it is common for nicotine-dependent smokers to also exhibit alcohol use disorders. Alcohol and nicotine both affect nAChRs, a shared substrate. Nicotine to alcohol: pre-exposure to nicotine increases alcohol cunsumption^26^; nicotine may prime VTA dopamine neurons to encode the reinforcing properties of ethanol more strongly^7^. Alcohol to nicotine: ethanol upregulates level of synaptic α4* nAChRs^27^; ethanol interacts with nAChRs, both directly and indirectly, in the mesocorticolimbic dopaminergic (DAergic) reward circuitry to affect brain reward systems^28^. We can infer the following: 1. Smoking increases the pleasure of drinking; 2. Drinking without smoking can reduce the withdrawal response, but it will induce the desire to smoke; 3. Smokers with alcohol use disorder will have a strong withdrawal response when deprived of cigarettes, and they may be at risk of higher nicotine dependent level. We can recommend the following to smokers during the period of cutting back on cigarettes or quitting: 1. Do not drink alcohol while using varenicline, as alcohol stimulates nAChRs to reduce the effect on quitting cigarettes; 2. If smokers have severe alcohol use disorder, it is recommended to consult a psychiatrist for joint diagnosis and treatment of the alcoholic smokers, in order to prevent the emergence of alcohol withdrawal symptoms and the failure of smoking cessation; 3. Smokers who have successfully quit smoking should never drink alcohol again, because alcohol stimulates the activation of nAChRs, which will make the smoker have desire to smoke again called relapse.

### Caffeine drink intake

Currently, caffeine-containing beverages include coffee, tea, cola or cocoa, energy drinks, etc. There are adenosine A2A-dopamine D2 receptor heteromers in the striatum region of the brain, which simultaneously produce adenosine and dopamine^18^. Caffeine not only amplifies the addictive and toxic effects of drugs of abuse, but it also sensitizes dopamine receptors to direct or indirect agonists^18^. When smoking and consuming a caffeinated beverage simultaneously, there are two possible outcomes: reduced adenosine and increased dopamine secretion. In addition, caffeine and nicotine are both metabolized by the human cytochrome P450 (CYP-450), family 2, subfamily A, polypeptide 6 (CYP2A6), which will result in a decreased rate of nicotine metabolism (Figure 2). We can deduce that: 1. Caffeine increases the pleasure of dopamine produced by nicotine. 2. Caffeine inhibits the metabolism of nicotine, resulting in the same amount of smoking required to maintain a high blood concentration of nicotine. We can recommend to smokers: 1. Do not consume caffeinated beverages while smoking; 2. Reduce the frequency of caffein consumption; if there is no addiction, discontinue the habit of drinking caffeinated beverages.

**Figure:**
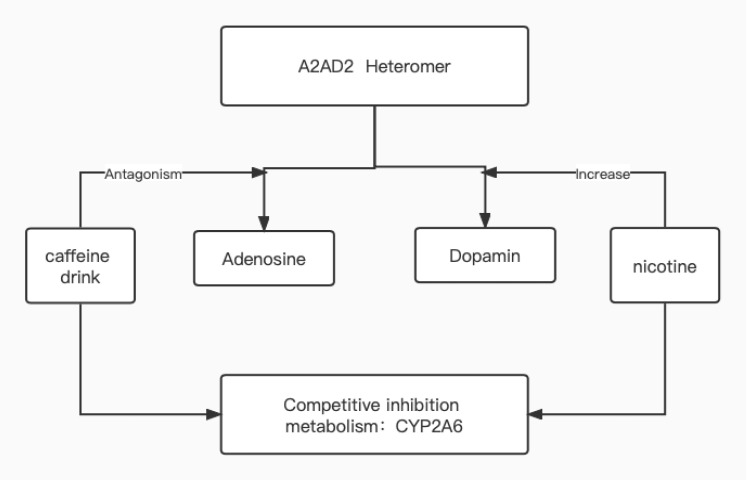
Picture 2

### Mental activities after dinner before sleep

Some mental activities induce dopamine consumption^29^: video games^30^, gambling^31^, overtime working^10^, playing chess, studying and so on. Due to their deficiency in dopamine, smokers would choose cigarettes as a supplement. More than one hour of mental activity after dinner will increase cigarette consumption 32. We recommend that smokers cease or reduce the time spent on mental activities after dinner that aid in smoking cessation.

### Physical exercise

Previous studies have confirmed that exercise have association with less smoking and lower nicotine dependence^9^, ^33^, ^34^. Aerobic exercise results in modifications to the mesolimbic pathway, which may mediate the exercise-induced suppression of drug-seeking behaviour 35. Physical activity can alleviate the depression brought on by withdrawal symptoms^36^. Consequently, moderate exercise is crucial during the period of smoking cessation.

### *CYP2A6* genotype

Nicotine is mainly metabolized by *CYP2A6* to inactivated cotinine^37^-^39^. *CYP2A6* genotype influences enzyme activity 40. CYP2A6*1 represents the normal rate of nicotine metabolism, whereas the majority of other gene loci represent a slow rate of nicotine metabolism 15. Smokers with a normal nicotine metabolism rate smoke more cigarettes 41, and smoking cessation rate is affected. Therefore, we require smokers to undergo *CYP2A6* genetic testing and provide recommendations based on test results: Smokers with the CYP2A6*1/ CYP2A6*1 genotype require a longer varenicline treatment duration or more time to quit smoking.

## Conclusion

Clinicians have the following recommendations for smokers without mental disorders during the period of smoking cessation:1. Do not use smoking to deal with your emotions; 2. Adequate duration and quality of sleep can help reduce smoking; 3. Do not consume alcohol or beverages containing caffeine while smoking (it is preferable to stop consuming alcohol and caffeine); 4. Do not engage in mental activities after dinner; 5. Increase exercise appropriately; 6. Based on the results of the CYP2A6 gene test, determine if it is necessary to extend the duration of treatment.

## References

[1] Burki TK (2021). WHO releases latest report on the global tobacco epidemic. Lancet Oncol.

[2] DeVita VT Jr (2005). The Framework Convention on Tobacco Control. Nat Clin Pract Oncol.

[3] Ayo-Yusuf OA (2005). WHO framework convention on tobacco control and its relevance to the dental professions in South Africa. SADJ.

[4] Silva LC, Araujo AJ, Queiroz AM, Sales MD, Castellano MV, Comissao de Tabagismo da S (2016). Smoking control: challenges and achievements. J Bras Pneumol.

[5] Karnath B (2002). Smoking cessation. Am J Med.

[6] McLaughlin I, Dani JA, De Biasi M (2015). Nicotine withdrawal. Curr Top Behav Neurosci.

[7] Morel C, Montgomery S, Han MH (2019). Nicotine and alcohol: the role of midbrain dopaminergic neurons in drug reinforcement. Eur J Neurosci.

[8] Ray R, Schnoll RA, Lerman C (2009). Nicotine dependence: biology, behavior, and treatment. Annu Rev Med.

[9] Strine TW, Okoro CA, Chapman DP, Balluz LS, Ford ES, Ajani UA (2005). Health-related quality of life and health risk behaviors among smokers. Am J Prev Med.

[10] Angrave D, Charlwood A, Wooden M (2014). Working time and cigarette smoking: evidence from Australia and the United Kingdom. Soc Sci Med.

[11] Chen A, Machiorlatti M, Krebs NM, Muscat JE (2019). Socioeconomic differences in nicotine exposure and dependence in adult daily smokers. BMC Public Health.

[12] Swanson JA, Lee JW, Hopp JW (1994). Caffeine and nicotine: a review of their joint use and possible interactive effects in tobacco withdrawal. Addict Behav.

[13] Hamidovic A, de Wit H (2009). Sleep deprivation increases cigarette smoking. Pharmacol Biochem Behav.

[14] Cohrs S, Rodenbeck A, Riemann D, Szagun B, Jaehne A, Brinkmeyer J (2014). Impaired sleep quality and sleep duration in smokers-results from the German Multicenter Study on Nicotine Dependence. Addict Biol.

[15] Lopez-Flores LA, Perez-Rubio G, Falfan-Valencia R (2017). Distribution of polymorphic variants of CYP2A6 and their involvement in nicotine addiction. EXCLI J.

[16] Shields PG, Herbst RS, Arenberg D, Benowitz NL, Bierut L, Luckart JB (2016). Smoking Cessation, Version 1.2016, NCCN Clinical Practice Guidelines in Oncology. J Natl Compr Canc Netw.

[17] Russell MA (1974). The smoking habit and its classification. Practitioner.

[18] Ferre S (2016). Mechanisms of the psychostimulant effects of caffeine: implications for substance use disorders. Psy-chopharmacology (Berl).

[19] Schultz W (1997). Dopamine neurons and their role in reward mechanisms. Curr Opin Neurobiol.

[20] Brischoux F, Chakraborty S, Brierley DI, Ungless MA (2009). Phasic excitation of dopamine neurons in ventral VTA by noxious stimuli. Proc Natl Acad Sci U S A.

[21] Holly EN, Miczek KA (2016). Ventral tegmental area dopamine revisited: effects of acute and repeated stress. Psychopharmacology (Berl).

[22] Chee MW, Choo WC (2004). Functional imaging of working memory after 24 hr of total sleep deprivation. J Neurosci.

[23] Thomas M, Sing H, Belenky G, Holcomb H, Mayberg H, Dannals R (2000). Neural basis of alertness and cognitive performance impairments during sleepiness. I. Effects of 24 h of sleep deprivation on waking human regional brain activity. J Sleep Res.

[24] Nilsson JP, Soderstrom M, Karlsson AU, Lekander M, Akerstedt T, Lindroth NE (2005). Less effective executive functioning after one night's sleep deprivation. J Sleep Res.

[25] Purani H, Friedrichsen S, Allen AM (2019). Sleep quality in cigarette smokers: Associations with smoking-related outcomes and exercise. Addict Behav.

[26] Tolu S, Marti F, Morel C, Perrier C, Torquet N, Pons S (2017). Nicotine enhances alcohol intake and dopaminergic responses through beta2* and beta4* nicotinic acetylcholine receptors. Sci Rep.

[27] Tarren JR, Lester HA, Belmer A, Bartlett SE (2017). Acute Ethanol Administration Upregulates Synaptic alpha4-Subunit of Neuronal Nicotinic Acetylcholine Receptors within the Nucleus Accumbens and Amygdala. Front Mol Neurosci.

[28] Hendrickson LM, Guildford MJ, Tapper AR (2013). Neuronal nicotinic acetylcholine receptors: common molecular substrates of nicotine and alcohol dependence. Front Psychiatry.

[29] Cools R, D'Esposito M (2011). Inverted-U-shaped dopamine actions on human working memory and cognitive control. Biol Psychiatry.

[30] Koepp MJ, Gunn RN, Lawrence AD, Cunningham VJ, Dagher A, Jones T (1998). Evidence for striatal dopamine release during a video game. Nature.

[31] Grant JE, Potenza MN (2005). Tobacco uses and pathological gambling. Ann Clin Psychiatry.

[32] Yu X, Yu Y, Ma H, Chen Z, Deng Z (2021). Mental activities after dinner increase cigarettes consumption. Sci Rep.

[33] Loprinzi PD, Walker JF (2015). Nicotine Dependence, Physical Activity, and Sedentary Behavior among Adult Smokers. N Am J Med Sci.

[34] Pokhrel P, Schmid S, Pagano I (2020). Physical Activity and Use of Cigarettes and E-Cigarettes Among Young Adults. Am J Prev Med.

[35] Robison LS, Swenson S, Hamilton J, Thanos PK (2018). Exercise Reduces Dopamine D1R and Increases D2R in Rats: Implications for Addiction. Med Sci Sports Exerc.

[36] He Q, Wu J, Wang X, Luo F, Yan K, Yu W (2021). Exercise intervention can reduce the degree of drug dependence of patients with amphetamines/addiction by improving dopamine level and immunity and reducing negative emotions. Am J Transl Res.

[37] McDonagh EM, Whirl-Carrillo M, Garten Y, Altman RB, Klein TE (2011). From pharmacogenomic knowledge acquisition to clinical applications: the PharmGKB as a clinical pharmacogenomic biomarker resource. Biomark Med.

[38] Benowitz NL, Hukkanen J, Jacob P (2009). Nicotine chemistry, metabolism, kinetics and biomarkers. Handb Exp Pharmacol.

[39] Porath AJ, Fried PA (2005). Effects of prenatal cigarette and marijuana exposure on drug use among offspring. Neurotoxicol Teratol.

[40] Satarug S, Tassaneeyakul W, Na-Bangchang K, Cashman JR, Moore MR (2006). Genetic and environmental influences on therapeutic and toxicity outcomes: studies with CYP2A6. Curr Clin Pharmacol.

[41] Malaiyandi V, Sellers EM, Tyndale RF (2005). Implications of CYP2A6 genetic variation for smoking behaviors and nicotine dependence. Clin Pharmacol Ther.

